# Biotin: a scoping review for Nordic Nutrition Recommendations 2023

**DOI:** 10.29219/fnr.v68.10256

**Published:** 2024-01-16

**Authors:** Beate Stokke Solvik, Tor A. Strand

**Affiliations:** 1 Department of Global Public Health and Primary Care, University of Bergen, Bergen, Norway; 2 Innlandet Hospital Trust, Lillehammer, Norway

**Keywords:** biotin, vitamin B7, carboxylation, nutrition recommendations

## Abstract

Biotin is a water-soluble B-vitamin with key roles in metabolism and are found in most foods at low concentrations. Symptomatic biotin deficiency is rare, and few studies have investigated biotin requirements in relation to health outcomes. Data to support the setting of dietary reference values for biotin are limited.

## Popular scientific summary

- Biotin, also known as vitamin B7, is a water-soluble vitamin with an essential role in fatty acid synthesis, gluconeogenesis, and catabolism of branched-chained amino acids.
- Biotin occurs in most foods, such as milk, liver, grains, yeast, soy, nuts, egg yolk, and some vegetables.
- A qualified biomarker of biotin status has not been identified.
- Deficiency, which is rare, can cause hair loss, conjunctivitis, scaly dermatitis, ataxia, hypotonia, seizures, and developmental delays.
- Dietary intake of biotin has not been estimated in national surveys in the Nordic population.

Biotin, also referred to as vitamin B_7_, is a water-soluble vitamin that is essential to all known organisms and is synthesized by plants and microorganisms. Animals and humans lack the ability to synthesize biotin and are therefore dependent on biotin from the diet (1). Biotin deficiency is rare as most foods contain biotin (2). Biotin functions as cofactors for several carboxylases that are involved in fatty acid synthesis, gluconeogenesis, and catabolism of branched-chained amino acids (2). In addition, biotin may have a role in cellular processes, including gene regulation (3). The aim of this scoping review is to describe the totality of evidence for the role of biotin for health-related outcomes as a basis for setting and updating dietary reference values (DRVs) for the Nordic Nutrition Recommendations 2023 (Box 1).

*Box 1.* The Nordic Nutrition Recommendations 2023 project.

- This paper is one of many scoping reviews commissioned as part of the Nordic Nutrition Recommendations 2023 (NNR2023) project (4).
- The articles are included in the extended NNR2023 report but, for transparency, these scoping reviews are also published in Food & Nutrition Research.
- The scoping reviews have been peer reviewed by independent experts in the research field according to the standard procedures of the journal.
- The scoping reviews have also been subjected to public consultations (see report to be published by the NNR2023 project).
- The NNR2023 committee has served as the editorial board.
- While these articles are a main fundament, the NNR2023 committee has the sole responsibility for setting dietary reference values in the NNR2023 project.

## Methods

This scoping review follows the protocol developed within the NNR2023 project (4).

The source evidence used in the scoping review follow the eligibility criteria described previously (5). The literature search was performed on February 2nd, 2022 in MEDLINE with the following search string: (“biotin”[MeSH Terms] OR “biotin”[All Fields]) AND review[Publication Type] AND (“2011”[PDAT] : “3000”[PDAT]) AND Humans[Filter]. The search strategy resulted in 217 publications. We screened the titles and abstracts of these publications and identified one study as potential eligible. This publication was not regarded as a qualified systematic review after reading the full manuscript (3). We also identified two reports from the European Food Safety Authority (EFSA) (6, 7). The EFSA report from 2012 (7) is regarded as a qualified systematic review (8). Additional relevant literature was found by using a snowballing approach.

## Physiology

### Bioavailability, digestion, absorption, transport, and excretion

Biotin in food exists in free and protein-bound forms, which differs between foods. For instance, most of the biotin in cereals and meats is protein-bound (9). In addition, synthesis of biotin by intestinal bacteria occurs in the large intestine, however, the contribution of this source to our biotin metabolism is unclear (2).

Free biotin is easily absorbed in the proximal small intestine mediated through the sodium-dependent multivitamin transporter (SMVT) (2). When biotin is consumed in pharmacological doses, the absorption occurs by passive diffusion and biotin is completely absorbed (10). The bioavailability of biotin in different foods varies mainly because protein-bound biotin requires digestion by biotinidase to release biotin prior to absorption (6). In addition, some compounds such as the glycoprotein avidin from raw egg whites binds biotin and prevents its absorption. The biotin-binding capacity of egg white is lost upon cooking (9).

In the blood, biotin is mainly transported as free biotin (81%) and to a lesser extent bound to plasma proteins (19%) (9). The uptake of biotin in peripheral tissues and the liver occurs via SMVT and the monocarboxylate transporter (MCT). Inside the cell, biotin is attached to biotin-dependent carboxylases. Limited storage of biotin occurs in the muscles, liver, and brain. Biotin and its metabolites are excreted via the urine and the non-absorbed biotin synthesized by intestinal bacteria is excreted via feces (11).

### Functions and mechanisms of biotin

Biotin functions as a cofactor for the enzymes acetyl-CoA carboxylase (ACC), β-methylcrotonyl-CoA carboxylase (MCC), pyruvate carboxylase (PC) and propionyl-CoA carboxylase (PCC). These enzymes are important for carboxylation reactions and assist in the transfer of one-carbon units in the form of activated carboxyl groups during intermediary metabolism. These reactions are also important in the synthesis of fatty acids, in the conversion of pyruvate into oxaloacetate (an intermediate in the citric acid cycle), and in the degradation of branched amino acids and odd-chain fatty acids (12). Moreover, biotin may have a non-coenzyme function in the human body, such as a role in gene regulation and genome stability (1, 3).

## Assessment of nutrient status

Urinary excretion of 3-hydroxyisovaleric acid (3HIA) and 3-hydroxyisovaleric acid-carnitine (3HIAc) are regarded as early and sensitive biomarkers of marginal biotin deficiency (13, 14). When biotin is sparse, these markers are products of alternative metabolic pathways for MCC. In addition, an abundance of lymphocyte holo-PCC and holo-MCC have been suggested to detect marginal biotin deficiency among healthy adults (3). However, the use of all the above-mentioned biomarkers has limitations due to large inter-individual variations and poor ability to distinguish between insufficiency and adequacy (6). As of today, there is no single biomarker that clearly distinguishes biotin-deficient versus biotin-sufficient individuals, and the dose-response relationships between biotin intakes, status, and biomarkers of biotin are unclear (6).

## Dietary intake in Nordic and Baltic countries

Biotin is found in most foods, such as milk, liver, grains, yeast, soy, nuts, egg yolk, and some vegetables at low concentrations (12). The dietary intake of biotin is not estimated in any of the national dietary surveys in the Nordic countries. Data on dietary intake of biotin in Latvia have been published in an EFSA report (6). In Latvia, estimations from two non-consecutive 24-h dietary recalls and a food frequency questionnaire from 1,377 adults (aged 17–65 years) showed a mean biotin intake between 34 and 45 µg/day. Moreover, this report also describes biotin intake from four other European countries (Austria, Germany, Hungary, and Ireland) (6). Among adults less than 65 years of age, the mean/median intake ranged from 26 to 50 µg/day, while for adults aged more than 65 years, the mean/median intake ranged from 24 to 43 µg/day.

In some European countries, estimation of biotin intake has also been done in children. In Germany and Ireland, the median biotin intakes were between 19 and 28 µg/day for children aged 1–4 years. In older children from Germany, Ireland, and Austria, the median or mean biotin intakes were between 19 and 38 µg/day, and 17 and 64 µg/day in children aged 5–12 and 13–19 years, respectively.

However, the accuracy of biotin estimations is questionable due to limitations of the food composition tables due to natural biotin variations of foods, and different analytical methods to determine the biotin content. Moreover, the countries’ dietary assessment methodologies to estimate the biotin intake also differed (5).

## Health outcomes relevant for Nordic and Baltic countries

Overt biotin deficiency is rare. Biotin deficiency has been reported in cases with total parental nutrition without biotin supplementation, on diets with chronic ingestion of raw egg white, or using certain antiepileptic medicines. Biotin deficiency has also been demonstrated in cases of inherited biotinidase deficiency (1). Symptoms of biotin deficiency include hair loss, conjunctivitis, scaly dermatitis, ataxia, hypotonia, seizures, and developmental delays in infants and children (9). The symptoms and consequences of suboptimal nutritional status (without overt deficiency) are uncertain (3). In several species, biotin deficiency during pregnancy is teratogenic, but no data exists to confirm this association of biotin deficiency during pregnancy and foetal malformation in humans (3).

Data on adverse effects from high biotin intake is insufficient to set a tolerable upper intake level (UL). Existing evidence from observational studies in other countries indicates that current levels of biotin intake from all sources do not represent a health risk for the general population (15).

## Requirement and recommended intakes

Data providing an estimate of biotin requirements are limited, and no DRVs were given in NNR2012 (16). EFSA proposes an adequate intake (AI) of 40 µg/day for adults based on observed mean/median biotin intakes from mixed diets (6). The biotin intake level is deemed adequate due to seemingly absence of biotin deficiency in these populations. The same AI for adults is also proposed for pregnant women. For lactating women, EFSA suggests an additional 5 µg/day, based on estimation of the human milk biotin content and infant consumption. For infants aged less than 6 months, an AI of 4 µg/day is proposed based on calculations of average human milk biotin content and infant consumption. An AI of 6 µg/day is suggested for infants above 6 months of age, this is based upon biotin intake of exclusively breastfed infants and extrapolated to older infants by reference body weight. For older children, the proposed AIs are based on the observed median biotin intake of each age group. The AI for children 1–3 years, 4–10 years, and adolescents (11–17 years) are set to 20, 25, and 35 µg/day, respectively (6). In an experimental study among pregnant and non-pregnant women with an average daily intake of approximately 60 µg biotin/day, pregnant women in the third trimester excreted more urinary 3HIA than the non-pregnant control women. The authors suggested that the biotin requirements in pregnancy exceed current AI, and that a biotin intake 2–3 times higher than the current AI is needed during pregnancy (17). However, more studies are needed to define the biotin requirements during pregnancy.

## Conflict of interest and funding

None
